# Traditional Sporting Games as an emotional induction procedure

**DOI:** 10.3389/fpsyg.2022.1082646

**Published:** 2023-01-06

**Authors:** Jorge Moya-Higueras, Jaume March-Llanes, Queralt Prat, Verónica Muñoz-Arroyave, Pere Lavega-Burgués

**Affiliations:** ^1^Department of Psychology, University of Lleida, Lleida, Spain; ^2^Centre for Biomedical Research Network on Mental Health (CIBERSAM), Instituto de Salud Carlos III, Barcelona, Spain; ^3^National Institute of Physical Education of Catalonia (INEFC), University of Lleida, Lleida, Spain; ^4^Motor Action Research Group (GIAM), Lleida, Spain; ^5^Institut de Recerca de Desenvolupament Social i Territorial (INDEST), University of Lleida, Lleida, Spain

**Keywords:** interpersonal relationships, motor praxeology, GES-II, ambivalent interactions, cognitive decisions

## Abstract

Experimental designs to induct emotional states have frequently used still procedures. However, more naturalistic methods of emotional induction by letting participants move and interact freely with other participants should be considered. Traditional Sporting Games (TSG) have the above-mentioned characteristics. The general aim of this study was to determine whether the different roles which allowed executing ambivalent interactions induced different emotional states in college students. We developed three studies with three paradoxical TSG (Sitting Ball Game, Four Corners Game, and Pitcher's Game). Before beginning to play, all the participants answered the Positive and Negative Affect Schedule (PANAS) in a mood version. After playing, participants were asked to report retrospectively the emotional state they were feeling in each role of the game, responding to the Self-Assessment Manikin, PANAS, and Games and Emotion Scale-II. Statistical analyses were performed by ANOVA, calculating corresponding effect sizes. Consistently, but specifically, in each game, roles still induced less positive and more negative emotions. Regarding the active roles, more positive and less negative emotions were kindled when the role allowed catching other players. On the contrary, when developing an active role that implied an increased likelihood of being caught, more negative and less positive emotions were experienced. We found some significant interaction effects between the moods and the role played before playing. To conclude, TSG could be an adequate procedure to induct emotional states and to study emotional conditions in a naturalistic way, showing ecological validity.

## Introduction

In natural contexts, the human emotional experience is a cause and a consequence of social interaction (Hari et al., 2015; Gilam and Hendler, 2016). Although emotions and social information do not share the same brain regions, there is an overlap in most of the brain structures (Gilam and Hendler, 2016). So, understanding how social interactions relate to emotions is a key objective of modern neuroscience (Gilam and Hendler, 2016; Panksepp et al., 2017).

Scientists developed several procedures to induct emotional states (Lang et al., 1999, 2008; Marchewka et al., 2014; Trost et al., 2017; Fernández-Aguilar et al., 2018; Geethanjal et al., 2018). Visual stimulation using pictures (Lang et al., 1999, 2008; Marchewka et al., 2014) or videos (Fernández-Aguilar et al., 2018) has been commonly used. Another source of emotional activation is hearing, with music being the primary stimulus used (Trost et al., 2017; Geethanjal et al., 2018). These procedures have been usually used in research because they allow the use of modern neuroscientific procedures, such as fMRI (Gilam and Hendler, 2016). However, they need the person being evaluated to be passive. For example, picture stimulation procedures (Lang et al., 1999, 2008; Marchewka et al., 2014) require seeing a series of pictures of emotional content but without any possibility of interacting with the picture designed with the stimuli that appear in them. The same can be said about the video (Fernández-Aguilar et al., 2018) or music (Trost et al., 2017; Geethanjal et al., 2018) induction procedures. More modern tasks, such as games, require some cognitive decisions that elicit emotional states (Gilam and Hendler, 2016), though the participant has to be physically motionless.

Developing a procedure to induce emotions with instructions to control different kinds of experiences in experimental conditions would allow testing the emotional experience in a more natural way. Traditional Sporting Games (TSG) have the abovementioned characteristics. Depending on the type of motor relationship, the theory of motor action or motor praxeology (Parlebas, 2001) introduces the concept of sociomotor games for TSG where players interact with peers and or opponents (e.g., fighting games and team games). Sociomotor TSG have original rules as a result of local tradition. Accordingly, some of these games activate a system of relationships very different from that of classic collective sports (Parlebas et al., 2016).

Furthermore, some TSG, such as the Sitting Ball Game (SBG; Lavega et al., 2018), allow ambivalent interactions. An ambivalent interaction leads participants into ambiguous or paradoxical situations where each player is potentially an ally and an opponent of the other players at the same time (Parlebas et al., 2016). Against this backdrop of contradictory relationships, it is hard to predict the players' behavior because each individual will act following his or her subjective socio-affective preferences at different times during the game (Obœuf et al., 2008). So, TSG dynamics could be a way to assess social interactions.

To the best of our knowledge, no study with a rigorous methodology has been undertaken yet to examine whether TSG is apt to induce emotional states or not. So, we performed three studies, with different TSG, to test the hypothesis that TSG promotes a significant change in the emotional state after playing them. All the games used in this study activated ambivalent interactions.

Based on the effect sizes obtained from the study of Lavega et al. (2017), the sample size was calculated for the three studies by assuming the following parameters: a design of repeated measures, a level of significance of 95% CI, a minimum desired power of 80%, considering two-tailed hypotheses and, a minimally-interesting effect size of 0.3. Calculations were made with the G^*^power 3.1.9.7 software. Considering 10% of possible losses due to errors in the registry, the objective sample size calculation was 99 subjects.

## Methodological overview

We designed three independent pre-post experiments to test whether TSG could promote a significant change in emotional experience after playing them. All the instruments regarding emotional experiences were assessed before and after participating in each TSG. According to the nature of the games, all the players acted in all possible roles. Games are adequately explained in each section. All the game sessions in the three studies lasted 8 min. In addition, all the TSG used were non-scoring games. During the game, participants constantly change their roles, and no rule marks the end of the game; the end of the game is marked by a standard duration of 8 min. This *ad-hoc* rule allowed us to compare better the emotional experiences through all the games. Finally, in the results section of each study, we first discuss the effects of playing the game and all the evoked emotions. Then, we focus on the different roles to test whether playing each role modulates the affective state.

## Study 1: Sitting ball game

### Materials and methods

#### Participants

We recruited 102 students (19 women and 83 men; aged between 18 and 26 years, *M*_*age*_ = 20.08 years, *SD* = 2.07) as participants from the University of Lleida. About 93.14% of the students had competitive sports experience (team sports). They were all first-year undergraduates pursuing a physical education and sports science degree. It should be noted that this study formed part of a training program for prospective physical education teachers, the aim of which was to raise their awareness about the relationship between motor intelligence and emotional intelligence. All students gave their active consent to participate. The research ethics committee of the University of Lleida approved the present study.

#### Instruments

One of the most used systems to assess emotional induction is the Self-Assessment Manikin (SAM; Lang et al., 2008; Moltó et al., 2013). However, Marchewka et al. (2014) explained that SAM was not a consistent measure of emotion because different populations could associate different semantics to each scale's extremes, especially for the arousal one. Moreover, finding an analogous emotional response in different rating systems should indicate that the result is consistent and should be less affected by measurement bias. For these reasons, we decided to assess emotional responses with three different scales.

The SAM is the first test to measure the dimensions of pleasure, arousal, and dominance using a series of abstract figures horizontally arranged according to a nine-point scale. Pleasure ranged from a frowning to a smiling figure, and arousal spanned from a relaxed, sleepy to an excited, wide-eyed figure, showing an incremental explosion at the center, while dominance ranged from a very small to a huge figure. The present test is widely used as it is a pictographic representation of emotional states and has shown good validity and reliability (Bradley and Lang, 1994; Moltó et al., 2013).

Another test to assess the intensity of emotions experienced specifically during games is the Games and Emotion Scale-II (GES-II; Lavega-Burgués et al., 2017). Participants responded on a seven-point scale to the level of intensity experienced for each of the five basic emotions (joy, sadness, anger, rejection, and fear). A score of one meant they had hardly felt that emotion, while a score of seven was indicative of maximum intensity. The present test was specifically designed to assess emotional experiences when doing physical exercise, showing good psychometrical properties (Lavega-Burgués et al., 2017).

Finally, we assessed mood before the games and the emotional state after finishing them with the Positive and Negative Affect Schedule (PANAS; Watson et al., 1988; Sandin et al., 1999). This measure consists of two ten-item mood scales to assess the Positive Affect (PA) and the Negative Affect (NA). Respondents were asked to rate the extent to which they experienced each particular emotion within a specified period with reference to a five-point scale (from 1 “very slightly or not at all” to 5 “very much”). We used the present questionnaire two times. The first one was to assess the mood state of the participants before the games' session, asking how they had felt “during the past 2 weeks.” Second, we also requested the emotional state “when they were developing each role of the game” to assess the emotional state linked to the game experience. Validation studies performed in Spain and other countries showed that PANAS is valid and reliable to assess emotional states (Watson et al., 1988; Sandin et al., 1999; Ortuño-sierra et al., 2015).

#### Procedure

Before beginning the game, participants answered the mood version of the PANAS. Then, they played the Sitting Ball Game (SBG). This game is a TSG found in some European countries. The rules allow ambiguous or paradoxical relationships since each player can decide whether he/she wants to cooperate with or oppose the other participants. When players are free (alive role) and have the ball, they may decide to pass it to another player with a bounce (a collaborative action) or through the air (an opposition action), capturing the target player who must sit down on the ground (prisoner role; Lavega et al., 2018). If the prisoner players intercept the ball, they return to the alive role (Guillemard et al., 1984). Therefore, this game has three strategic roles: alive with the ball, alive without the ball, and prisoner (Lavega et al., 2018). During the game, each player has the autonomy to decide to collaborate or to oppose in any situation. Each decision is a relationship and also involves an emotional experience. The decisions and the emotional states of the participants in SBG put into action two interconnected realities: the internal logic of the game (system), which activates internal relations between the players, and the social actors (Scheve and Luede, 2005; Lavega et al., 2014). The adaptation of the players to internal logic leads each person to decide whether he/she is going to lead cooperative or oppositional relationships, i.e., improvise strategies associated with alliances and unpredictable betrayals. When the game ended, we asked the participants to rate their emotional state (with the SAM, GES, and PANAS) by retrospectively remembering how they felt when developing each role during the game.

#### Statistical analysis

After the initial descriptive exploration, the general linear model was carried out several times to evaluate the effect of role (within-subject factor), explaining the observed differences between dimensions of GES, SAM, and PANAS questionnaires. ANOVAs and ANCOVAs were applied to test factors and covariates introduced in each model. The analyses were performed with the SPSS package 24.0 (IBM Corp, 2016).

### Results

ANOVAs revealed significant changes in all emotional outcomes [from *F*__*Rejection*−*GES*_(2, 202)_ = 9.90, *p* < 0.001, $\eta_{p}^{2}$ = 0.16 to *F*__*Valence*−*SAM*_(2, 202)_ = 97.81, *p* < 0.001, $\eta_{p}^{2}$ = 0.66] with the exception of the domination scale of the SAM [*F*_(2, 202)_ = 2.80, *p* = 0.066, $\eta_{p}^{2}$ = 0.05]. After controlling the effect of mood before the game, the results remained the same [from *F*__*Rejection*−*GES*_(2, 198)_ = 9.93, *p* < 0.001, $\eta_{p}^{2}$ = 0.17 to *F*__*Valence*−*SAM*_(2, 198)_ = 96.21, *p* < 0.001, $\eta_{p}^{2}$ = 0.66]. Full results can be seen in Table 1.

**Table 1:** Mean, standard deviations, ANCOVAs, and planned contrasts of Sitting Ball emotional induction game.

	**Alive with the ball (A)**			**Alive without the ball (B)**			**Prisoner (C)**			***F***	** $\eta_{p}^{2}$ **	**Planned contrasts**			
**Sense co-variants**	***M***	***CI***		***M***	***CI***		***M***	***CI***				***F* (A vs. B)**	** $\eta_{p}^{2}$ **	***F* (A vs. C)**	** $\eta_{p}^{2}$ **
Joy/positive	5.12	4.84	5.40	3.82	3.55	4.10	2.85	2.55	3.16	82.18^***^	0.63	90.94^***^	0.48	160.23^***^	0.62
Sadness	1.32	1.20	1.45	1.78	1.56	2.01	3.01	2.68	3.34	51.78^***^	0.51	16.28^***^	0.14	104.47^***^	0.51
Fear	1.46	1.26	1.67	3.03	2.64	3.42	1.41	1.25	1.58	37.10^***^	0.43	61.80^***^	0.38	0.13	0.00
Anger	2.29	1.96	2.63	1.88	1.61	2.15	2.83	2.44	3.23	15.70^***^	0.24	6.24^*^	0.06	6.51^*^	0.06
Rejection	1.60	1.36	1.84	1.78	1.54	2.03	2.44	2.09	2.80	9.93^***^	0.17	1.69	0.02	17.94^***^	0.15
Neg Emo	6.68	6.07	7.28	8.48	7.68	929	9.70	8.70	10.69	17.24^***^	0.26	20.64^***^	0.17	34.07^***^	0.26
VAL	2.29	2.00	2.59	3.82	3.52	4.13	5.53	5.14	5.92	96.21^***^	0.66	94.34^***^	0.49	192.55^***^	0.66
INT	3.32	2.99	3.66	3.78	3.41	4.14	5.28	4.92	5.63	32.48^***^	0.40	7.19^**^	0.07	65.49^***^	0.40
DOM	5.76	5.33	6.18	5.29	4.90	5.69	5.56	5.16	5.96	2.80	0.05	4.72^*^	0.05	0.53	0.01
PA	35.21	33.80	36.62	31.01	29.57	32.45	27.15	25.56	28.73	64.44^***^	0.57	80.11^***^	0.45	122.69^***^	0.55
NA	15.28	14.46	16.09	19.82	18.49	21.16	18.06	16.96	19.16	35.82^***^	0.42	68.44^***^	0.41	31.70^***^	0.24

M, Estimated marginal means. Controlling for mood (PANAS) before playing the game.

* p < 0.05;

** p < 0.01;

*** p < 0.001.

$\eta_{p}^{2}$ < 0.06, small effect size; 0.06 < $\eta_{p}^{2}$ < 0.14, medium effect size; $\eta_{p}^{2}$ > 0.14, large effect size.

When comparing the emotional scores reported within the different roles (see Table 1), playing alive with the ball generated more positive emotions than the other roles, measured with GES and with PANAS (compared to alive without the ball, *p* < 0.001, and prisoner, *p* < 0.001). Regarding negative emotions, GES revealed that the prisoner role elicited more sadness (*p* < 0.001), more anger (*p* < 0.001), and more rejection (*p* < 0.001) than any other role. On the contrary, when players were alive without the ball, they experienced more fear than in any other situation (*p* < 0.001). The general GES scale of negative emotions showed a higher score when in the prisoner role (*p* < 0.001), while the negative affect scale (PANAS) showed a higher score when participants were alive without the ball. The results of the SAM scale were in accordance with the GES, reporting higher negative valence (*p* < 0.001) and higher intensity (*p* < 0.001) when playing the prisoner role.

## Study 2: Four corners game

### Materials and methods

#### Participants

We recruited 119 students (21 women and 98 men; aged between 18 and 28 years, *M*_*age*_ = 21.01 years, *SD* = 2.27) as participants from the University of Lleida. About 77.7% of the students had competitive sports experience (team sports). They were all 1st-year undergraduates pursuing a physical education and sports science degree. It should be noted that this study formed part of a training program for prospective physical education teachers, the aim of which was to raise their awareness about the relationship between motor intelligence and emotional intelligence. All students gave their active consent to participate. The research ethics committee of the University of Lleida approved the present study.

#### Instruments

The instruments of the present study were the same as study 1.

#### Procedure

Consistent with study 1, we administered the PANAS mood version to the participants before they began to play the Four Corners Game. This TSG is found in many European and American countries. This game is played in a square space of about 5 × 5 m. Five players can participate in the game, of which one is located in the center (center role) and the rest in each of the four corners (corner role). The players in the corners try to change corners at their will, avoiding the player in the center arriving before them. The player who is left without a corner goes on to occupy the center. The rules allow the existence of ambiguous or paradoxical relationships since each player can decide whether he/she wants to cooperate or oppose the participants of the other corners. In this game two players cooperate when they agree to exchange their corners, synchronizing the actions. Two players oppose each other when, after deciding to go out to exchange the position, one of them shows that he/she is going to go out or goes out a few meters and then returns to his/her corner. In that circumstance, the partner of another corner is in a situation of ambivalent relation that the center role player takes advantage of to occupy that corner. Once all the participants ended playing, the SAM, GES, and PANAS were administered retrospectively while the participants were asked to request to remember how they felt when developing each role during the game.

#### Statistical analysis

A similar strategy was applied as used in study 1.

### Results

The ANOVAs revealed significant changes in all emotional outcomes [from *F*__*Dominance*−*SAM*_(1, 87)_ = 6.66, *p* = 0.12, $\eta_{p}^{2}$ = 0.07 to *F*__*Positiveemotion*−*GES*_(1, 87)_ = 114.52, *p* < 0.001, $\eta_{p}^{2}$ = 0.57] with the exception of the intensity scale of the SAM [*F*_(1, 87)_ = 2.48, *p* = 0.112, $\eta_{p}^{2}$ = 0.03]. After controlling the effect of mood before the game, the intensity scale of the SAM remained non-significant [*F*_(1, 85)_ = 2.58, *p* = 0.112, $\eta_{p}^{2}$ = 0.03] and the fear scale of the GES became non-significant [*F*_(1, 85)_ = 0.90, *p* = 0.345, $\eta_{p}^{2}$ = 0.03] while the other results remained significant. Full results are shown in Table 2.

**Table 2:** Mean, standard deviations, ANCOVAs, and planned contrasts of Four Corners emotional induction game.

	**Corner (A)**			**Center (B)**			***F***	** $\eta_{p}^{2}$ **	**Interaction with PA (mood)**		**Interaction with NA (mood)**	
**Sense co-variants**	***M***	***CI***		***M***	***CI***				***F***	** $\eta_{p}^{2}$ **	***F***	** $\eta_{p}^{2}$ **
Joy/positive^*^cov	4.55	4.26	4.83	2.82	2.54	3.10	126.224^***^	0.60	0.95	0.01	10.49^**^	0.11
Sadness^*^amb cov	1.49	1.31	1.67	2.31	2.02	2.59	36.42^***^	0.30	14.57^***^	0.15	0.34	0.00
Fear^*^amb cov	1.77	1.52	2.03	1.64	1.42	1.85	0.90	0.10	0.37	0.00	3.74	0.04
Anger^*^amb cov	1.60	1.37	1.83	2.14	1.84	2.43	14.71^***^	0.15	2.23	0.03	0.91	0.01
Rejection^*^amb cov	1.44	1.29	1.60	2.10	1.81	2.40	19.04^***^	0.18	0.12	0.00	1.31	0.02
Neg Emotion^*^cov	6.31	5.79	6.82	8.18	7.39	8.97	30.79^***^	0.27	6.55^*^	0.07	0.50	0.01
VAL^*^amb cov	2.65	2.34	2.95	4.64	4.22	5.05	83.88^***^	0.50	3.91	0.04	1.44	0.02
INT^*^amb cov	4.02	3.67	4.38	3.72	3.30	4.13	2.58	0.03	5.21^*^	0.06	0.20	0.00
DOM^*^amb cov	5.65	5.28	6.01	5.13	4.75	5.51	6.81^*^	0.07	3.62	0.04	0.16	0.00
PA^*^amb cov	30.27	29.00	31.54	27.68	26.20	29.16	28.05^***^	0.25	4.04^*^	0.05	0.80	0.01
NA^*^amb cov	16.02	15.14	16.91	19.28	18.08	20.49	41.21^***^	0.33	4.30^*^	0.05	2.40	0.03

M, Estimated marginal means; PA, Positive affect; NA, Negative affect.

* p < 0.05;

** p < 0.01;

*** p < 0.001.

$\eta_{p}^{2}$ < 0.06, small effect size; 0.06 < $\eta_{p}^{2}$ < 0.14, medium effect size; $\eta_{p}^{2}$ > 0.14, large effect size.

In this game, each participant could be at the corner or the center. As the game has only two roles, the means could be directly compared through the significant ANOVA results. Hence, the corner role showed more positive emotions and fewer negative emotions than the center role.

**Figure 1:**
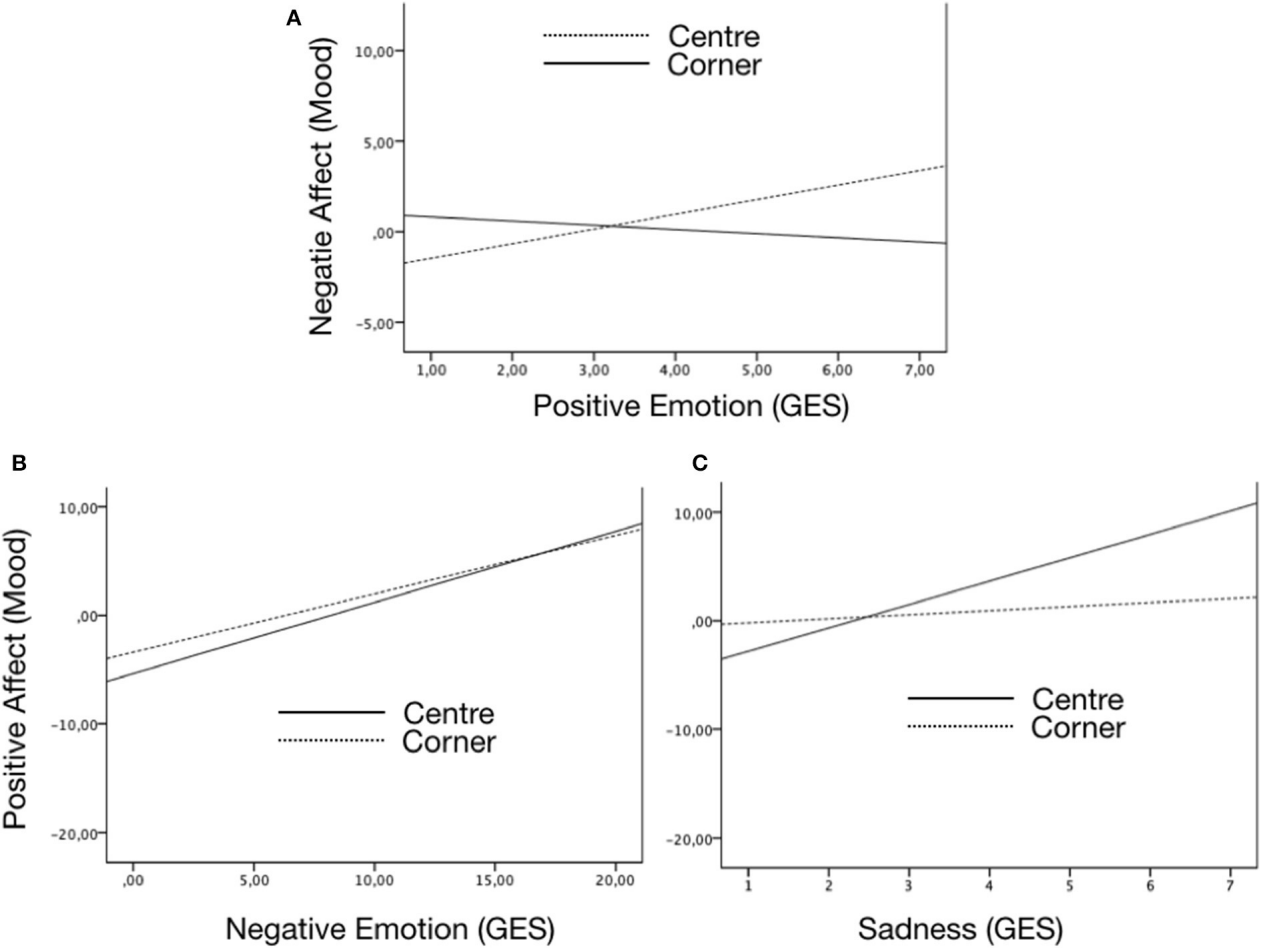
Significant interactions between emotions induced (by the GES) and mood (PANAS) in the Four Corner game. **(A)** Positive Emotion (GES) and Negative Affect (PANAS). **(B)** Negative Emotion (GES) and Positive Affect (PANAS). **(C)** Sadness (GES) and Positive Affect (PANAS).

In the robust analysis, mood was introduced as a factor in the ANOVAs to control its effect. In this game, some interactions were found between the initial mood and the game's role in different emotions (see Figure 1). For example, participants with higher negative affect (mood) experienced more positive emotions when they were at the center than when they were at the corner. On the other side, participants with higher positive affect felt more negative emotion and, especially, higher sadness when they were at the center than when they were at the corner.

## Study 3: Pitcher's game (elbow game)

### Materials and methods

#### Participants

We recruited 129 students (35 women and 94 men; aged between 18 and 27 years, *M*_*age*_ = 20.99 years, *SD* = 2.23) as participants from the University of Lleida. About 78.29% of the students had competitive sports experience (team sports). They were all 1st-year undergraduates pursuing a physical education and sports science degree. It should be noted that this study formed part of a training program for prospective physical education teachers, the aim of which was to raise their awareness about the relationship between motor intelligence and emotional intelligence. All students gave their active consent to participate. The research ethics committee of the University of Lleida approved the present study.

#### Instruments

The instruments of the present study were the same as studies 1 and 2.

#### Procedure

We administered the PANAS mood version to the participants before they began playing the Pitcher's Game. This is a TSG that has been played since the middle ages in different European countries. The players are placed in pairs joined by one arm. Each pair is separated from the other pairs at about 2 m. All pairs delimit the playing field that represents a big circle. These players share the role of the pitcher. There are also two other players, with the roles of Cat and Rat. The Cat chases the Rat, and if he/she manages to touch the Rat, the roles are exchanged. The Rat moves where it wants, either inside the circle or behind the pitchers. When the Rat joins a person in the pitcher role, the person on the other side of the pitcher must abandon the role of a Pitcher and go out assuming the Rat role. In this game, ambivalent relationships arise from the moment a Rat player decides which Pitcher is going to become the next Rat (opposition relationship) by joining his/her partner or allowing him/her to continue in the role of a Pitcher (cooperation relationship) by taking the arm of that player. In this game, the changes in the roles derive from contradictory relations of cooperation and opposition without any apparent logic. Similar to previous studies, participants answered the SAM, GES, and PANAS, recollecting how they felt when developing each role during the game.

#### Statistical analysis

A similar strategy was applied as used in studies 1 and 2.

### Results

In this game, significant results were found in all emotional outcomes [from *F*__*Rejection*−*GES*_(2, 232)_ = 3.14, *p* = 0.037, $\eta_{p}^{2}$ = 0.05 to *F*__*PositiveAffect*−*PANAS*_(2, 232)_ = 43.15, *p* < 0.001, $\eta_{p}^{2}$ = 0.45]. All results remained significant after controlling the effect of mood [from *F*__*Rejection*−*GES*_(2, 208)_ = 3.11, *p* = 0.048, $\eta_{p}^{2}$ = 0.05 to *F*__*PositiveAffect*−*PANAS*_(2, 208)_ = 42.29, *p* < 0.001, $\eta_{p}^{2}$ = 0.45]. Full results can be seen in Table 3.

**Table 3:** Mean, standard deviations, ANCOVAs, and planned contrasts of Pitchers' emotional induction game.

	**Cat (A)**			**Rat (B)**			**Pitcher (P)**			***F***	** $\eta_{p}^{2}$ **	**Planned contrasts**			
**Sense co-variants**	***M***	***CI***		***M***	***CI***		***M***	***CI***				***F*** **(A vs. B)**	$\eta_{p}^{2}$	***F*** **(A vs. C)**	$\eta_{p}^{2}$
**Amb co-variants**															
Joy/positive	4.11	3.79	4.43	4.66	4.38	4.93	4.50	4.23	4.78	5.11^**^	0.08	10.19^**^	0.08	4.14^*^	0.04
Sadness	1.83	1.59	2.07	1.39	1.22	1.56	1.37	1.23	1.51	8.32^***^	0.13	13.51^***^	0.11	24.80^***^	0.12
Fear^a^	1.59	1.38	1.79	2.58	2.25	2.91	2.24	1.95	2.53	19.73^***^	0.26	34.51^***^	0.23	21.70^***^	0.16
Anger	2.04	1.76	2.31	1.41	1.25	1.57	1.31	1.17	1.44	19.15^***^	0.25	31.72^***^	0.22	36.38^***^	0.24
Rejection	1.70	1.49	1.92	1.44	1.24	1.63	1.42	1.24	1.60	3.11^*^	0.05	4.96^*^	0.04	4.70^*^	0.04
Neg Emotion	7.17	6.47	7.87	6.82	6.21	7.43	6.35	5.81	6.88	3.65^*^	0.06	1.28	0.01	6.69^*^	0.06
VAL	3.59	3.23	3.95	3.02	2.68	3.35	3.19	2.86	3.51	5.77^**^	0.09	11.55^**^	0.09	3.41	0.03
INT	3.42	3.08	3.75	2.93	2.55	3.31	4.94	4.59	5.30	28.13^***^	0.33	18.66^***^	0.14	36.50^***^	0.24
DOM	5.31	4.95	5.68	5.17	4.77	5.58	5.80	5.43	6.18	3.93^*^	0.07	0.82	0.01	5.98^*^	0.05
PA	33.72	32.57	34.88	35.50	34.27	36.72	28.09	26.75	29.44	42.29^***^	0.45	10.95^**^	0.10	58.84^***^	0.36
NA	18.37	17.26	19.49	19.77	18.50	21.03	17.44	16.42	18.47	12.44^***^	0.18	11.60^**^	0.09	4.89^*^	0.04

M, Estimated marginal means.

a Interaction between the positive affect mood factor and the roles' factor.

* p < 0.05;

** p < 0.01;

*** p < 0.001.

$\eta_{p}^{2}$ < 0.06, small effect size; 0.06 < $\eta_{p}^{2}$ < 0.14, medium effect size; $\eta_{p}^{2}$ > 0.14, large effect size.

*Post-hoc* analyses of the three roles (see Table 3) revealed that the Rat role induced more positive emotional states than the Cat role (Joy/positive_SAM_, *p* = 0.002, Valence_SAM_, *p* = 0.001, Positive Affect_PANAS_, *p* = 0.001). Regarding negative emotions, the results are not so consistent comparing different roles. While we found higher mean scores on Negative Affect_PANAS_ (*p* = 0.001) for the Rat role, the Negative Emotion scale of the GES revealed higher scores (but not significant) when assuming the Cat role (*p* = 0.37). The valence scale of the SAM was more similar to the GES than to the PANAS scale. Focusing on specific emotions, participants showed higher levels of fear when in the Rat role (*p* = < 0.001) but higher levels of sadness (*p* = < 0.001), anger (*p* = < 0.001), and rejection (*p* = 0.028) when in the Cat role.

**Figure 2:**
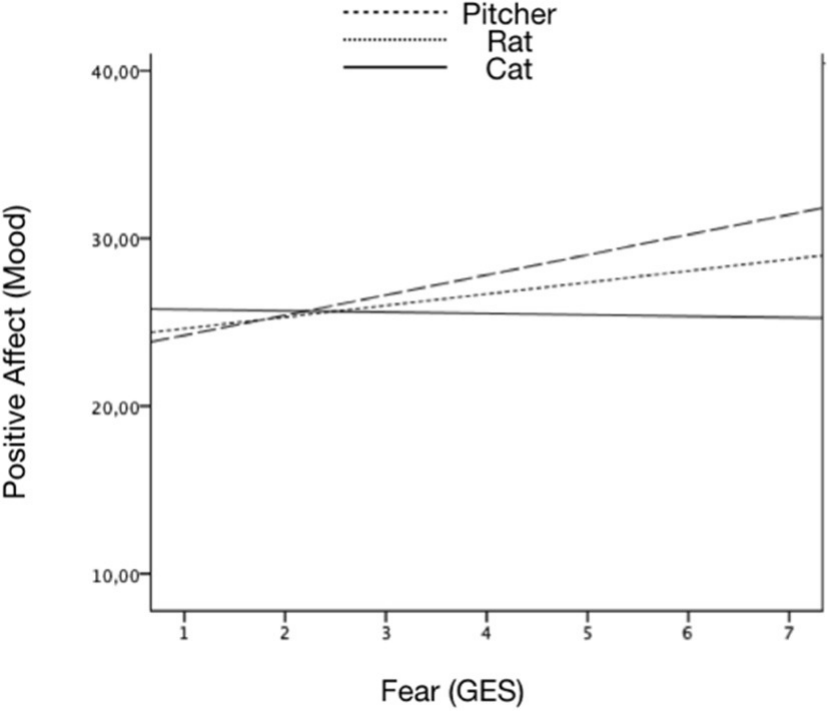
Significant interactions between emotions induced (by the GES) and mood (PANAS) in the Pitcher game.

Finally, in line with the Four Corners Game, we found a significant interaction between mood and role factors. Specifically, those participants with positive moods experienced the most fear in the Pitcher role and the least in the Cat role (see Figure 2).

## General conclusion

The main objective of the present study was to examine whether playing TSG with ambivalent interactions generates different emotions or not. Ambivalent TSG means that each player could change the role he/she is developing according to the game's rules but in an unpredictable way because of the interaction with others (Parlebas et al., 2016).

According to the results of the studies performed with three different TSGs, the hypothesis of the study was confirmed. Moreover, to test the hypothesis, we assessed emotional states with three different measures (SAM, PANAS state, and GES). The same kind of emotional state was replicated across the different measures after controlling the effect of mood at the baseline. We also found some significant interaction effects between moods before beginning to play TSGs and reported emotions with specific roles. These results showed the importance of controlling the effect of the previous mood in emotion induction procedures as past studies showed (Fernández-Aguilar et al., 2018). In addition, the present results are in line with previous initiatives of capturing emotional changes while playing TSG (Lecrosiey, 2017). In this study, the authors showed that facial expressions changed when players acted in different roles. While the study of Lecrosiey (2017) focused on directly monitoring the emotional states while playing, we emphasized the subjective emotional experience. Both studies point out that, when playing TSG, different roles derive specific emotional experiences.

As we used the SAM system, we could compare the magnitude of activation of TSG with other emotion induction procedures. Valence and intensity scores were lower in the present study than using pictures (Marchewka et al., 2014), though it was similar to using films (Fernández-Aguilar et al., 2018). Marchewka et al. (2014) explained that they used different descriptions for the scales' extremes, though the original studies also showed higher valence and arousal scores with pictures than what we found with TSG (Moltó et al., 1999, 2013). On the other hand, there are significant differences in our procedure compared to using other kinds of stimuli. In the present study, in all the TSG, participants played an entire match, and then, they estimated their emotions when performing each possible role, retrospectively. When using pictures or films (Moltó et al., 1999; Marchewka et al., 2014; Fernández-Aguilar et al., 2018), participants were questioned immediately after watching the stimuli. Hence, we hypothesize that if we could stop the game and ask the participants when they have finished each role, we could find higher valence and intensity ratings. However, this procedure would completely change the dynamics of the game, making it impossible to pursue an experimental design like the present one. Nevertheless, future studies could focus on assessing emotional responses more immediately when using TSG. One possible solution could be incorporating physiological recordings. The main problem with this suggestion is that participants must move to play TSG. When registering physiological responses in lab settings, people are calm and relaxed, and they are usually explicitly incited to not move (Levenson, 2014). Eliciting emotions with TSG is incompatible with staying calm, relaxed, and static. Although wearable wireless biosensors are still in their first steps (Salim and Lim, 2019), future studies of emotion induction in natural settings, such as playing TSG, could use these kinds of instruments.

As the present study focused on basic emotions, the specific feelings derived from the different roles in the games are in line with different theoretic emotion models (Ekman and Cordaro, 2011; Izard, 2011; Levenson, 2011; Panksepp and Watt, 2011). For example, in the SBG (Guillemard et al., 1984; Lavega et al., 2018), players felt more fear when they were alive without the ball than in any other role, while they felt more sadness when they were prisoners than in any other role. In the SBG, when a player is free but he/she has not got the ball (alive without the ball), there is a chance of receiving the ball through the air. If the player does not intercept the ball and the ball touches him/her, then he/she becomes a prisoner. As a prisoner, players do not interact in the game because they are forced to sit down unless they intercept the ball (which is very difficult). Hence, as fear becomes evident when we detect a threat (Ekman and Cordaro, 2011), players in the alive without the ball role are threatened by the possibility of a change in their status to a prisoner role. Similar to when we are overcome with sadness when we lose anything (Ekman and Cordaro, 2011), the players playing the prisoner role, who have lost the chance of playing actively in the game, in this game are also overcome with sadness. Similar results were found in the other two games.

We believe that these results could be different if we study secondary or social emotions. All the basic emotion models conclude that one difference between basic and non-basic emotions is the magnitude of appraisal (Tracy and Randles, 2011). While automatic appraisals characterize basic emotions, non-basic emotions depend more on elaborated ones. The concept of appraisal could be understood as to how a person interprets the stimulus or situation (Lazarus, 1991; Phillips et al., 2003). Hence, it will depend on how players interpret what is occurring during the game to feel one emotion or another. For example, in the SBG, if a player feels that two other players are always collaborating between them but opposing him/her, the player could feel jealous of not being included in the collaborating strategy of the other two players. Alternatively, the player could feel hate because the other two players are against him/her. It could be even possible to feel both emotions at the same time. Nevertheless, these are hypothetical results that we have not studied in the present research. In addition to retrospectively asking for the emotion felt in each role, we could ask for the general appraisal of the situation. So, in future studies, we expect to develop this perspective.

The present study has some limitations. An intrinsic characteristic of TSG is in itself a limitation, i.e., when participants are playing continuously while adopting different roles, we could not stop the game each time they assumed a new role to ask them about the emotion they were feeling in each role. The assessment of the emotion induction was performed retrospectively after completing the game. In more usual procedures, such as picture or movie induction, the stimuli are presented, and shortly thereafter (milliseconds or seconds), a subjective response is requested (Moltó et al., 1999; Marchewka et al., 2014; Fernández-Aguilar et al., 2018). Another limitation was that all the participants were undergraduate students from physical education and sports majors. It is possible that the results might be different with students of other degrees or with young adults of the general population. Besides, Fernández-Aguilar et al. (2018) found significant age differences in mood induction procedures. So, it could be expected that older people playing TSG would report different emotional states than younger participants. Finally, though the sample size was adequate in the last two studies, the sample size in the first study was limited. It would be advisable to replicate the present study with bigger samples.

Thus, to conclude, we presented three studies with different TSG. Consistently and according to the internal logic of the game, as players developed each role, they felt different emotional states. The results were mostly consistent with three different measures of emotional states. So, we propose to use TSG in future studies to assess emotional responses and states in a more naturalistic fashion.

## Data availability statement

The raw data supporting the conclusions of this article will be made available by the authors, without undue reservation.

## Ethics statement

The studies involving human participants were reviewed and approved by Ethics Committee of the University of Lleida. The patients/participants provided their written informed consent to participate in this study.

## Author contributions

JM-H and JM-L led the writing of the manuscript and the statistical analyses. QP and VM-A helped with finishing the manuscript, reviewing the different drafts, and in the execution of the project. PL-B coordinated the tea, designed the study, and was in charge of collecting the data. All authors contributed to the article and approved the submitted version.
